# *Isatis tinctoria* L.—From Botanical Description to Seed-Extracted Compounds and Their Applications: An Overview

**DOI:** 10.3390/plants14152304

**Published:** 2025-07-25

**Authors:** Justine Dupré, Nicolas Joly, Romain Vauquelin, Vincent Lequart, Élodie Choque, Nathalie Jullian, Patrick Martin

**Affiliations:** 1 Unité Transformations & Agroressources, Université d’Artois—UniLaSalle, ULR7519, F-62408 Béthune, France; 2 BioEcoAgro-Biologie des Plantes et Innovation, UMRT INRAe 1158 BioEcoAgro, Université de Picardie Jules Verne, F-80039 Amiens, France

**Keywords:** *Isatis tinctoria* L., woad, Brassicaceae, seed, chemical composition, fatty acids, amino acids, phytosterols, glucosinolates

## Abstract

*Isatis tinctoria* L. (Brassicaceae), also known as woad or dyer’s woad, is an ancient plant with a rosy future ahead. Most of the knowledge about woad is related to indigo dye production and its medicinal applications, especially its leaves. The general interest in woad has decreased with the rise of petroleum-based products. However, nowadays this plant is attracting interest again with industries reintroducing natural dyes. To meet the market demand in a sustainable manner, recent studies have focused specifically on woad seeds, leading to a valorization of the whole woad plant. This review provides an overview of the botanical, phytochemical composition, and properties of woad seeds, primarily supporting their cosmetic and pharmaceutical potential. From a chemical point of view, woad seeds mainly contain fatty acids, amino acids, phytosterols and glucosinolates. These compounds have been investigated through their extraction and analytical methods, as well as their properties and industrial applications.

## 1. Introduction

*Isatis tinctoria* L. (Brassicaceae), also known as woad, is native to Southeastern Russia and is located throughout Europe, North Africa, Asia and North America [1,2,3]. The plant’s cultivation and use have undergone many changes over the centuries. Woad was cultivated in Europe for many centuries during the Middle Ages [4,5,6,7]. *Isatis tinctoria* L. was originally a source of blue dye [3,4,5,6,8,9,10,11,12,13], and to a lesser extent, a paint component and an active ingredient in medicines [9,12,13] from the 12th to the 17th century. Its fermented rosette leaves were a source of indigo and used for luxury dyeing [3,11,12,13]. Over time, the woad industry has encountered two crises and has been replaced by other indigo sources, which offer higher recovery yields, brighter colors, and lower costs [12,13,14]. Woad cultivation first competed qualitatively and quantitatively with imported East India indigo during the seventeenth century [4,5,12,14,15]. Quantitatively, tropical *Indigofera tinctoria* had a five times better yield extraction (0.2% against 1%), which increased again with synthetic methods, especially the Baeyer–Drewsen [2,16] (1882), the Steingruber [17] (2004) and then the Steingruber [17] (2004) methods [13]. At the end of the 19th century, plant-derived indigo was neither competitive nor well understood compared to the synthetic one [4,14,15,18]. Therefore, the cultivation of *Isatis tinctoria* L. for indigo production declined, giving way to synthetic indigo involving a more profitable process and a chemically indistinguishable pigment [4,9,12,14,18]. Thus, the cultivation and uses of woad have been forgotten and are only growing in wild places such as roadsides or random lands [3,11,13,19]. Moreover, woad became problematic because of its invasive characteristics, especially in the USA [19,20]. After a century, with a view to returning to naturalness, woad crops and other indigo-producing crops have been recently reintroduced by growers [4,9,13] for natural dye production [4,9,10,15,18]. A zero-waste approach has been recently adopted for an overall recovery of *Isatis tinctoria* L., including branches, flowers, leaves, roots, and to a lesser but not insignificant extent, seeds and oil [13,21,22]. Some research laboratories, notably in the Hauts-de-France region, are involved in this global study of woad. The aim of such research is to guarantee the long-term re-establishment of this plant in the region, through high value-added applications. The present review will begin with the phenology and botanical characteristics of the woad to better understand the life cycle of the plant and the specifications of its fruits. In the second part, the review will compare the compounds of *Isatis tinctoria* to those of a similar species, *Isatis indigotica* Fort. Indeed, it allows us to see the differences and the similarities between the two *Isatis* species, and therefore put forward hypothetical woad molecules. Then, the cosmetic and pharmaceutical properties of each molecule listed in the literature for woad seeds are studied, with a view to promoting their use in these fields. The extraction and analysis processes are also described, with particular attention paid to energy costs and extraction process efficiency. Next, concentrations of compounds in *Isatis tinctoria* seeds will be highlighted and compared with those in *Isatis indigotica*, for each molecular family. Finally, focus will be placed on the antioxidant activity to enhance the wellness potential of woad fruits and seeds.

## 2. Phenology and Botanical Description

### 2.1. International Names and Botanical Situation

*Isatis tinctoria* L. is the scientific botanical name of the plant, but it can be called by different names throughout the world, even in the same country. These vernacular names are listed in Table 1.

To complete this overview, it is important to remember the botanical situation of the plant studied. Indeed, the species *Isatis tinctoria* L. belongs to the *Isatis* genus and to the Brassicaceae (Cruciferous) family [23]. In fact, plants with similar botanical situations, especially those of the same genus, may have similar chemical contents and/or similar activities and properties.

### 2.2. Botanical Description of I. tinctoria L. Fruit

**Figure 1:**
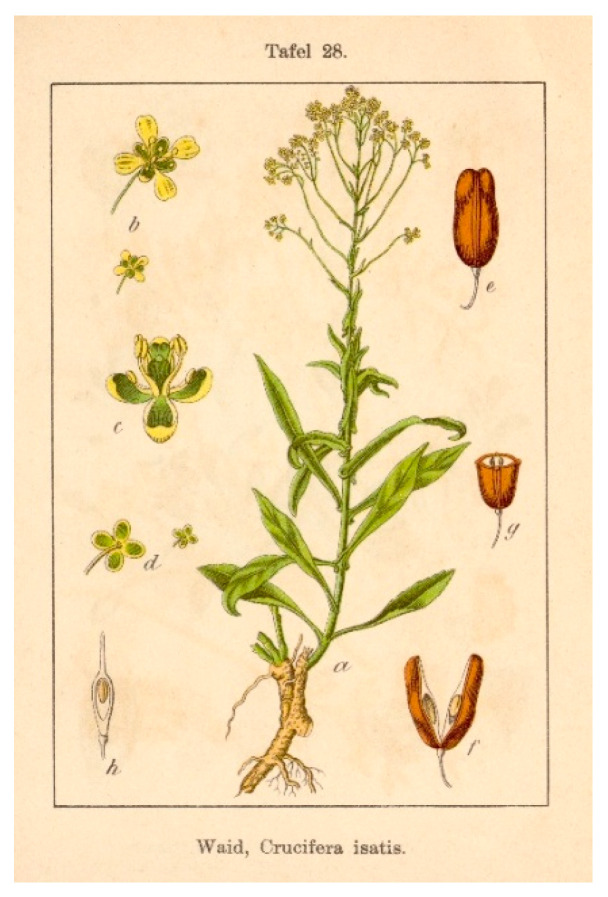
Botanical drawing of *Isatis tinctoria* L. [26].

*Isatis tinctoria* L. is a biennial plant that produces fruit every two years. The whole fruit is composed of a fruiting pedicel, a pod, a pericarp, and a seed (Figure 1).

The fruiting pedicel is 5–10 mm long [3,11]. This ramification between the stem and the fruit is slender and subclavate at apex [3].

The silicle is an indehiscent and winged pear-shaped fruit [12,23,27]. The protective silicle is composed of a woody pod which encases the pericarp and the seed [3,12,19]. The pendulous seed pod turns from light green to blackish-brown when mature [11,22,28]. The hairless or shortly hairy fruit is 10–20 mm long, 3–6 mm wide, with apical wings 3.5–5 mm wide [3,11,22,27,28]. The silicle shape is qualified as oblong, obovate, oblanceolate and elliptic with a cuneate base [3,22,27]. It has a distinct midvein and inconspicuous lateral veins [3,11]. The yellowish-brown seed is 2.3–3.5 mm long and 0.8–1 mm wide [3,11,12]. It is an uniovulated ovary, narrowly oblong, and encircled by a pericarp [23].

### 2.3. Phenology and Germination

The phenology and the germination of *Isatis tinctoria* L. are described in Table 2, in order to gain a better understanding of its life cycle.

**Figure 2:**
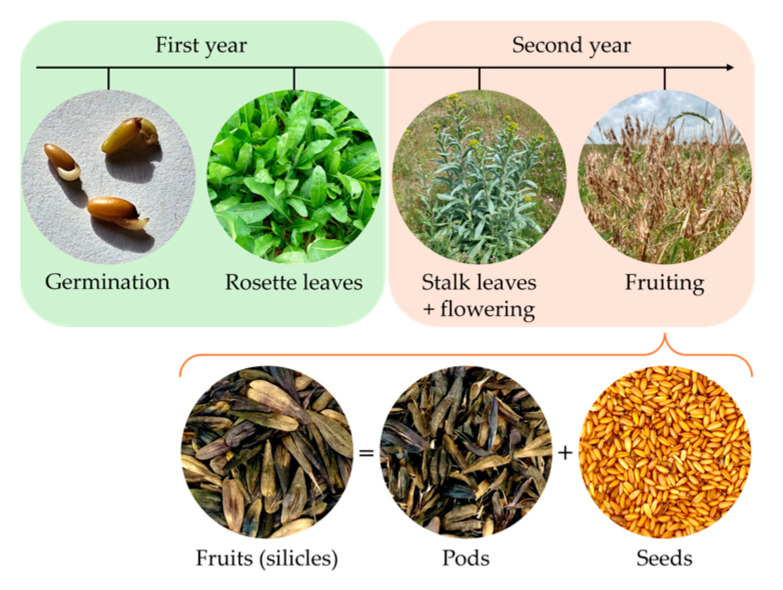
*Isatis tinctoria* growth stages.

### 2.4. Reproduction

Fuller [12] described that different modes of reproduction are possible for woad. On the one hand, the rosette can reproduce asexually from the tap root. On the other hand, the stalk (or bolted) form can reproduce asexually, from the roots, or sexually, leading to seeds. Woad seed reproduction does not exactly follow the Brassicaceae pattern. Indeed, seeds germinate quickly and plenty of seedlings develop, as in typical Brassicaceae. However, the fruits are indehiscent and naturally encapsulated by a germination inhibitor. This inhibitor blocks the germination of competing species, notably varieties of grasses and forbs such as other Brassicaceae.

Woad is known for its rapid growth and prolific seed production [20]. Such easy proliferation of this invasive species can lead to problematic management [19,20]. According to Farah et al. [20], the main factor in the spread of woad seed does not appear to be wind, but is strongly linked to the fruit pedicel, which behaves like a hook and adheres to objects, suggesting animal dispersal.

## 3. Comparison of the Overall Chemical Composition of *Isatis tinctoria* and *Isatis indigotica* Seeds

Four main molecular families have been investigated in woad seeds throughout the literature. All molecules identified in the seeds are listed in Table 3 and classified according to their chemical classes.

Each molecular family will then be described in detail. Only organic compounds will be studied in this review, although the microelement composition of woad seeds has been described by Kizil et al. [29].

Moreover, *Isatis tinctoria* and *Isatis indigotica* were long considered to be the same species, but there is now tangible evidence to the contrary [36]. Thus, a quick study of the phytochemical description of *Isatis indigotica* seeds will be presented in this review in order to link to other hypothetical molecules found in *Isatis tinctoria* seeds. Indeed, similar plants like these two may have similar phytochemical composition and properties. Nevertheless, authors of this review will focus only on molecules identified and proven in *Isatis tinctoria* seeds, and not on theoretical ones.

## 4. Fatty Acids

**Figure 3:**
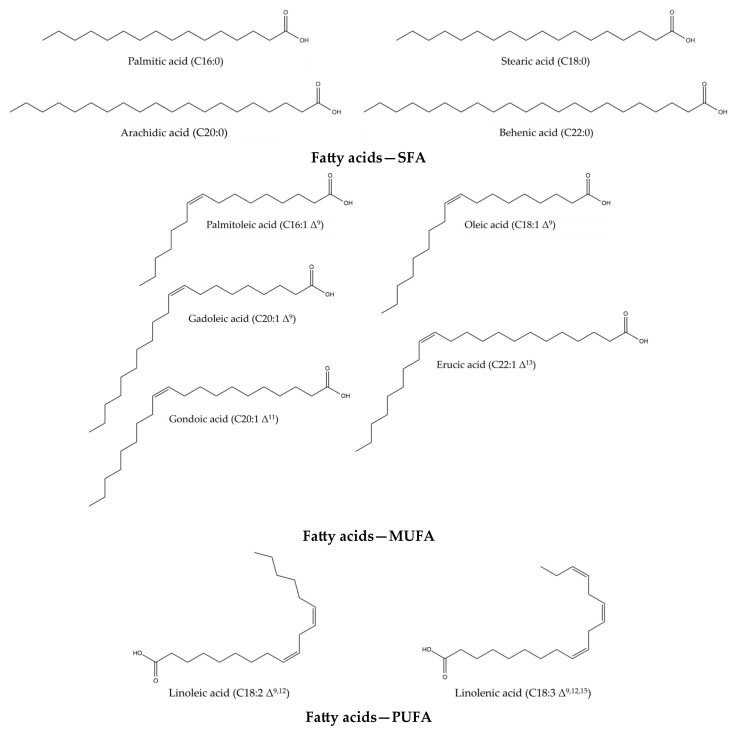
Structures of the fatty acid compounds in *Isatis tinctoria* seeds.

*Isatis tinctoria* contains 10 fatty acids (Figure 3) described in the literature. Of these, 4 are saturated fatty acids (SFAs), four are monounsaturated fatty acids (MUFAs) and two are both polyunsaturated fatty acids (PUFAs) and essential fatty acids for humans. *Isatis indigotica* also contains these fatty acids, but not only.

### 4.1. Functions and Properties of Fatty Acids

PUFAs can be synthetized by humans, but not essential fatty acids like linoleic acid and linolenic acid. As mentioned Iba [37], essential fatty acids must be incorporated through a diversified diet, to reach the amounts essential for each individual organism. Gillingham et al. [38] have highlighted the health protective feature of MUFAs. Indeed, a diet enriched with MUFAs prevents or improves metabolic syndrome and the risk of cardiovascular disease, by favorably modulating blood lipids, blood pressure and insulin sensitivity. Thus, the quality and the quantity of dietary fats affect the risk of cardiovascular diseases [37,39]. MUFAs have slightly lower or equivalent effects to those of PUFAs in reducing low-density lipoprotein cholesterol and total cholesterol in plasma [38]. Nevertheless, MUFAs improve high-density lipoprotein cholesterol levels and may have hypotensive effects. Furthermore, preferential oxidation and metabolism of MUFAs have an impact on body composition and may reduce the risk of obesity [38]. In addition, when dietary saturated fatty acids are replaced with carbohydrates, MUFAs are efficient at maintaining high-density lipoprotein cholesterol levels, lowering triglyceride levels and enhancing insulin sensitivity, which could help people with metabolic syndrome or diabetes mellitus [38]. Furthermore, a deficiency in PUFAs can lead to a reduction in prostanoid production and a deterioration in the epidermal barrier. On the other hand, too high a level of PUFAs can also be problematic, inducing high cholesterol levels, high blood pressure, high platelet aggregation capacity, and hence cardiovascular diseases.

Some of the fatty acids listed below have interesting properties, notably in cosmetics. For instance, Spataro and Negri [40] have demonstrated emollient and moisturizing properties for use in soaps and body creams. Indeed, cutaneous applications of PUFAs have been shown to protect the skin barrier and fix skin lesions, in the manner of an emollient agent. The properties of SFAs, MUFAs, and PUFAs described in Table 4 focus mainly on cosmetic properties and, to a lesser extent, on pharmaceutical and food properties.

### 4.2. Oil Extraction and Analyses of Its Fatty Acid Contents

Studies providing fatty acid content mentioned different procedures for oil extraction, fatty acid preparation and analysis. These methods and, to a lesser extent, complementary ones will therefore be discussed.

For rigorous fatty acids analysis, some precautions must be taken for sample preparations. Firstly, the whole fruit must be dried to 0% moisture content. Then, the seeds are extracted from their silicle and pericarp, either manually or mechanically. Next, seeds are grinded using a blade mixer, to obtain different particle sizes [9,29,50].

Although several oil extraction techniques are described in the literature for fatty acids extraction, only Soxhlet [9,29,31,32,51] and Ultrasound-Assisted Extraction (UAE) [31] were chosen by the authors who provided the fatty acid contents reported in the present study.

The conventional Soxhlet method is an accessible technique but has significant disadvantages. Soxhlet extraction continuously recycles the extractive solvent but is also time-consuming (2 h–8 h). As a result, this method requires high to moderate energy consumption due to the constant and prolonged, but not excessive, heating of the solvent. Also, Soxhlet extraction is applicable at high temperatures and boosts process kinetics, but the technique has a low extraction efficiency [31,52].

The UAE technique uses high-frequency sound waves to promote cavitation and fatty acid extraction. This technique requires a moderate amount of energy, particularly for generating ultrasound over a prolonged period.

Many other extraction techniques could have been described, such as cold-pressed extraction [53,54,55], acid hydrolysis extraction (method 95.402 of the Association of Official Analytical Chemists international (Official methods of analysis, 16th edn. AOAC, Arlington), Accelerated Solvent Extraction (ASE) [52], or maceration coupled with UAE [50,52]. We note that Romdhane and Gourdon [50] used maceration coupled with ultrasound to extract fatty acids from woad seeds, but no concentrations of these fatty acids were mentioned in their publication.

Consequently, we can rank all these extraction methods in ascending order of energy costs: (1) cold-pressed extraction; (2) UAE; (3) maceration combined with ultrasounds; (4) solid-liquid extraction; (5) Soxhlet; (6) ASE. Indeed, each technique has specific advantages, but thermal and pressure-based methods (ASE, Soxhlet) tend to have higher energy costs than room-temperature methods such as maceration and solid-liquid extraction.

Once the oil has been extracted from the biomass, two sample preparation steps must be followed. The first one consists of evaporating the solvent from the extract. The objective of this step is to concentrate oil extracts by totally removing the solvent using a rotary evaporator or a nitrogen stream [9,29], then analyzing them by Gas Chromatography (GC) [9,50].

The second step, as performed by Romdhane and Gourdon [50], consists of converting fatty acids into fatty acid methyl esters (FAMEs) for gas chromatography analysis. The authors mentioning the fatty acid contents of woad seeds used TBME method [9] and transmethylation [29,32], but there are several methods for converting fatty acids into their corresponding methyl esters, such as the BF_3_ method, the BHT method [29], and many other transesterification [56,57] and esterification methods [58]. The choice of method depends mainly on the composition of the oil. In fact, some methods are generally more efficient, others are recommended for samples with low acidity, and still others for samples with high acidity. To conclude, there are various methods for converting fatty acids into volatile methyl esters, including some that are not mentioned in this review. The choice must be made by considering simultaneously the composition of the oil, the fatty acids of interest, the necessary reagents, and the availability of equipment.

To calculate the average oil content of seeds, samples can be characterized gravimetrically and by GC with different types of detectors, such as Flame Ionization Detector (FID), Mass Spectrometry (MS), and Thermal Conductivity Detector (TCD). The authors who provided the fatty acid contents utilized GC-FID [9,32], GC-MS [29,31] and GC-TCD [51] methods, enabling accurate quantitative determination. To choose the most suitable method, it is necessary to consider the availability of equipment, standards and/or mass spectrometry libraries. Moreover, GC-MS and GC-FID methods are the most widely described techniques in the literature for fatty acid analysis.

Besides the composition of the oil itself, oil can be characterized in many ways, as mentioned by Gambert et al. [59] For instance, color and clarity can be determined visually, the refraction index is identified with a refractometer, and density is calculated with a pycnometer. Viscosity is studied with a rotary viscometer, calculating the torsional force, the speed of the axis, and its characteristics. The moisture content is calculated after a period of heating using a thermo-balance. Numerous indexes are registered to characterize oils. (1) The acid index (NF EN 14104 for fatty acid methyl esters) refers to the amount of free fatty acids. The free acid content of fats increases over time, making it a good indicator of their state of deterioration. (2) The total acid number determines the sum of all acid compounds (ASTM D664-18e2 for petroleum products). (3) The saponification index (NF EN ISO 3657 for fats; ASTM D94-07(2017) for petroleum products) gives the molecular weight of all medium-chain fatty acids present in a sample. (4) The iodine index (NF EN ISO 3961 for fats; NF EN 14111 for fatty acid methyl esters; ASTM D5554-15(2021) for fats and oils) is used to assess the level of unsaturation in the oil. (5) The peroxide index (NF EN ISO 3960 for fats; ASTM E299-17a for organic solvents) is utilized to calculate the oxygen level in the oil.

### 4.3. Fatty Acid Composition in Isatis tinctoria Seed Oil

Table 5 compares the ratio of fatty acids from *Isatis tinctoria* and *Isatis indigotica* seeds according to crop and production site. 

It appears that the contents of all SFAs, MUFAs, and PUFAs varies according to these different sites. The Turkish site quantified higher amounts of palmitic acid (SFA) than the French and US sites for *Isatis tinctoria*. Higher quantities of C20:1 fatty acid (MUFA) were found at the US site than at the other sites for *Isatis tinctoria*. The Turkish site had low quantities of erucic acid (MUFA) compared to the other two countries for *Isatis tinctoria*. Linoleic and linolenic acids (PUFAs), which are essential fatty acids, were found in low quantities at the Turkish site compared to those in France and the USA for *Isatis tinctoria*.

Roche et al. [9] and Mikolajczak et al. [51] showed that unsaturated fatty acids (MUFAs and PUFAs) are predominant in the composition of *Isatis tinctoria* seed oil. This trend is also confirmed by Li et al. [31] and Angelini et al. [32] for *Isatis indigotica* seed oil. Also, Kizil et al. [29] pointed out that MUFAs are the main class of fatty acid in woad oil. By cross-referencing data from different production sites, the main compounds in *Isatis tinctoria* and *Isatis indigotica* oils are oleic acid, erucic acid, and linolenic acid. The predominance of these three fatty acids is a common characteristic of the Brassicaceae oils [37]. These fatty acids can be useful in the cosmetics industry due to their emollient, surfactant, and fragrance properties. However, a cautionary note must be issued as the erucic acid content of the seeds greatly exceeds the amount permitted for food use. This amount must therefore also be controlled for cosmetic or pharmaceutical applications. Varietal selection limiting the erucic acid content within the plant could be a sustainable solution to the toxicity issue of this molecule, while complying with the regulations governing its potential applications.

The physico-chemical characteristics of the oil, based on the results of Dolya et al. [30], are summarized in Table 6. 

According to Iba’s thesis [37], the fatty acid composition, particularly with a high concentration of unsaturated fatty acids, provides specific physical properties. Indeed, low viscosity is explained by the high amount of unsaturated fatty acids. The high density implies an oil with quite high saponification and iodine indexes. The refractive index is induced by the presence of unsaturated fatty acids with long carbons chains. However, as mentioned above, the fatty composition depends on the production site, the harvest period and the harvest year. Thus, the values presented in Table 6 are not necessarily constant for *Isatis* seed-extracted oil.

The refractive index is similar for both *Isatis tinctoria* oil and *Isatis indigotica* oil. *Isatis tinctoria* oil has a lower acid index, i.e., a lower free fatty acid content, than *Isatis indigotica* oil. The saponification index provides information on the average molecular weight of all bound and free fatty acids present in a sample. *Isatis tinctoria* oil has a higher saponification index, i.e., a lower molecular weight of all medium-chain fatty acids, than *Isatis indigotica* oil. *Isatis tinctoria* oil has a higher iodine index, i.e., more unsaturations or double bounds, than *Isatis indigotica* oil.

## 5. Amino Acids

**Figure 4:**
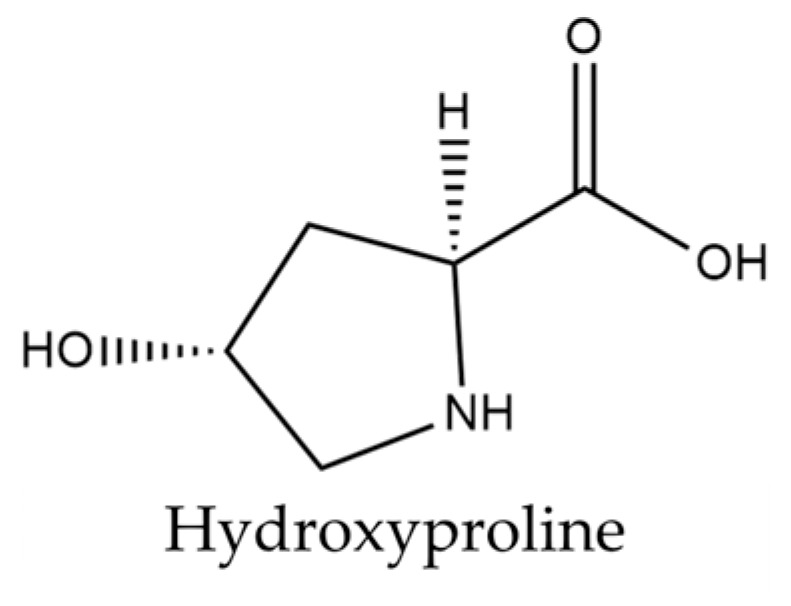
Structure of hydroxyproline present in woad seeds.

*Isatis tinctoria* contains 18 amino acids described in the literature. *Isatis indigotica* also contains these amino acids, except for hydroxyproline. Only hydroxyproline is shown in Figure 4, as the other amino acids described are relatively common.

### 5.1. Functions and Properties of Amino Acids

In general, amino acids play a crucial role in the structure, metabolism and physiology of cells, as they are the building blocks of peptides and proteins. In a study, Wu et al. [60] described that amino acids have significant biological, nutritional and health functions. In addition, amino acids are cell signaling molecules; they are key precursors for proteins, including hormones and low-molecular-weight nitrogenous compounds. Amino acids are also regulators of metabolic pathways and processes, which are essential for health, such as growth, development, reproduction, immunity, and homeostasis of living organisms. For instance, they contribute to the regulation of gene expression and protein phosphorylation cascades. Nevertheless, physiological concentrations of amino acids and their products (e.g., nitric oxide, polyamines, glutathione, taurine, thyroid hormones, and serotonin) are necessary for proper metabolic function. Too high levels of these substances can lead to neurological disorders, oxidative stress, and cardiovascular disease. An optimal balance of amino acids in dietary supplementation is essential for good health. Amino acids are valuable for improving health at different stages of life, for example in cases of fetal growth retardation, neonatal morbidity and mortality, intestinal dysfunction and weaning-related wasting syndrome, obesity, diabetes, cardiovascular disease, metabolic syndrome, infertility, and infection. They are also beneficial for enhancing the efficiency of metabolomic modifications to improve muscle protein synthesis, muscle growth, milk production, egg and meat quality, and athletic performance, while preventing excess fat deposition and reducing adiposity in living beings.

A total of 17 of the 20 basic amino acids are referenced for *Isatis tinctoria*. Hydroxyproline, formed from proline by hydroxylation, is the only derived molecule described in the literature for this plant. Although hydroxyproline is not specific to *Isatis* and is not the most abundant amino acid, this molecule remains interesting. Kumar Srivastava et al. [61] explained that its two isomeric forms (trans-4-hydroxy-L-proline and trans-3-hydroxy-L-proline) play a key role in the synthesis and thermodynamic stability of collagen fiber, suggesting a strong potential for hydroxyproline in cosmetic application. These two molecules are found in animal collagens, which are qualified as structural proteins.

VanEtten et al. [62] highlighted that hydroxyproline is found in the seed pod and pericarp of the woad fruit. It can therefore be assumed that structural proteins are present in these two parts of the plant. The protective property of the woad pod may be, to some extent, due to the presence of these proteins. The amino acids present in woad seeds have interesting cosmetic and pharmaceutical applications, which are summarized in Table 7.

### 5.2. Extraction and Analyses of Amino Acids

The research conducted by Miller et al. [33] focused on all amino acids in the seed pod of the woad, while VanEtten et al. [62] concentrated on hydroxyproline content in both the pod and the pericarp. With regard to sample preparation, seeds were grounded and then extracted with petroleum ether to remove the oil [33,62]. Next, the samples are acid-hydrolyzed before analysis [33,62,64]. 

**Figure 5:**
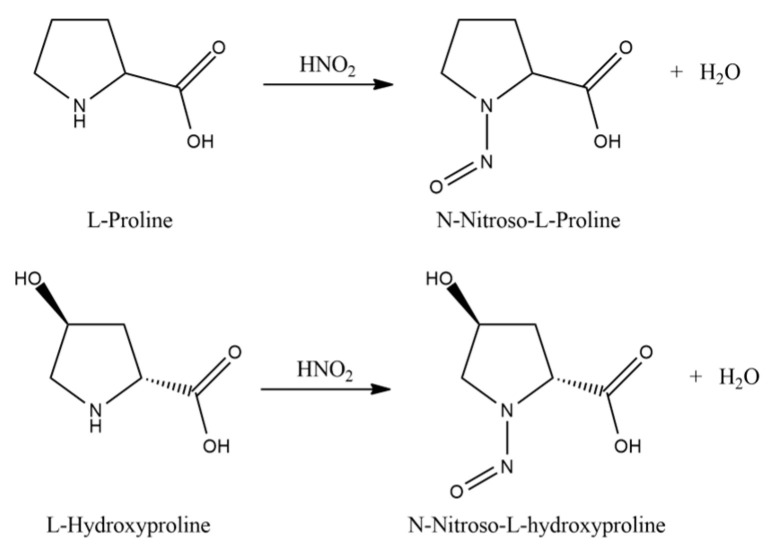
Reaction of the acid hydrolysis of L-proline and L-hydroxyproline.

Then, amino acids are isolated from the acid hydrolyzate of the seed pod using a procedure similar to that described by Levine [65]. The two imino acids of L-proline and L-hydroxyproline undergo acid hydrolysis by heating with nitrous acid (Figure 5). 

Low-Pressure Liquid Chromatography (LPLC) separates the two nitrosoamines thus obtained. Once crystallized, L-hydroxyproline is analyzed by X-ray fluorescence spectrometer (XRF) and its optical rotation is calculated. Furthermore, the sample elementary analysis gives C, 45.7%; H, 6.96%; N: 10.5%. The theoretical one gives C, 45.7%; H, 6.87%; N, 10.68%.

VanEtten et al. [62] highlighted the following two methods for quantitative amino acid analysis. The first one is the Ion Exchange Chromatography (IEC) method developed by Spackman, Stein and Moore [66] using an MS instrument and an UV detector at 440 nm. The second one is a Thin-Layer Chromatography (TLC) method, which refers to the method developed by Becker, Milner and Sagel [67].

### 5.3. Amino Acid Composition

Miller et al. [33] determined the overall amount of amino acids found in woad seeds harvested in the USA (Table 8). 

On a dry basis and after acid hydrolysis, I. tinctoria contains 12.5% c of crude protein in the whole seed and 14.3% c in the extracted meal. This species contains 12.6% of oil, also on a dry basis. In seed meals, 69.8% of the total nitrogen was accounted for as amino acids. Moreover, VanEtten et al. [62] calculated 150 mg of hydroxyproline per gram of nitrogen in *Isatis tinctoria*, confirmed by Miller et al. [33] who found 148 mg of this molecule per gram of nitrogen. The most abundant amnio acids in woad seeds are glutamic acid and, to a lesser extent, aspartic acid.

## 6. Phytosterols

**Figure 6:**
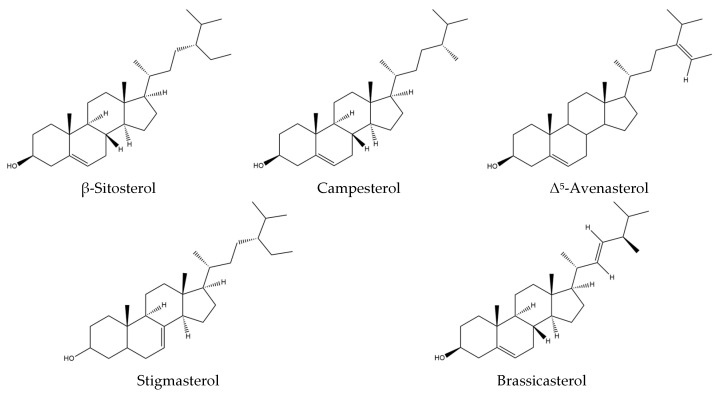
Structures of the phytosterol compounds in *Isatis tinctoria* seeds.

*Isatis tinctoria* contains seven phytosterols described in the literature, including two found in trace amounts (Figure 6). Phytosterols are currently not described in *Isatis indigotica*.

### 6.1. Functions and Properties of Phytosterols

Phytosterols have applications in everyday life. Indeed, they add value to crop yields as well as human nutrition, pharmaceutical, and cosmetic applications [9]. Consuming phytosterols significantly decreases serum cholesterol levels [68,69,70,71,72,73,74,75], which lowers the risk of cardiovascular diseases [69,72,73,74,75]. Thanks to phytosterol-enriched diets, the toxic effects of these compounds enable tumoral cell apoptosis [68,72,74,75]. This molecular property helps to reduce the risk of common cancers such as lung, stomach, colon, breast, and prostate cancers [68,69,72,74,75]. In addition, Burg et al. [76] have shown that mixtures of phytosterols, mainly composed of stigmasterols, may be useful in preventing Alzheimer’s disease [68]. Other therapeutic properties have been reported, such as anti-inflammatory, anti-bacterial, anti-ulcerative, angiogenic, immunomodulatory, antidiabetic, anti-tumoral, antinociceptive, antituberculosis, and anti-atherosclerotic ones [68,69,73,74]. To summarize, Plaza et al. [77] emphasized that food and dietary supplements enriched with phytosterols offer many health benefits. In a less well-documented way, phytosterols also have cosmetic properties [41,68]. Table 9 summarizes all the properties mentioned above, focusing mainly on the cosmetic and pharmaceutical ones. However, some compounds have no described applications in cosmetics, probably due to a lack of tested biological activities. 

### 6.2. Extraction and Analyses of Phytosterols

Phytosterols are contained in woad-extracted oil but cannot be separated from it as they are. Indeed, the samples need to undergo a saponification step (for phytosterol esters) and a derivatization (silylation) step. Finally, sterol trimethylsilyl ether derivatives can be analyzed by GC [9,83].

### 6.3. Phytosterol Composition

The authors want to point out that the phytosterols in woad seeds are not very well described in the literature, explaining why few references are available, and even fewer recent ones.

With regard to phytosterol composition, Roche et al. [9] studied phytosterol compounds according to their quantity encountered in woad seeds and their site of collection (Table 10). 

The main phytosterol found in woad seeds is β-sitosterol, which accounts for more than 50% of the total sterol content. The second main phytosterol is campesterol, representing 20% of the sterol content. The third most abundant one is Δ^5^-avenasterol, accounting for approximately 15% of the sterol content.

## 7. Glucosinolates

**Figure 7:**
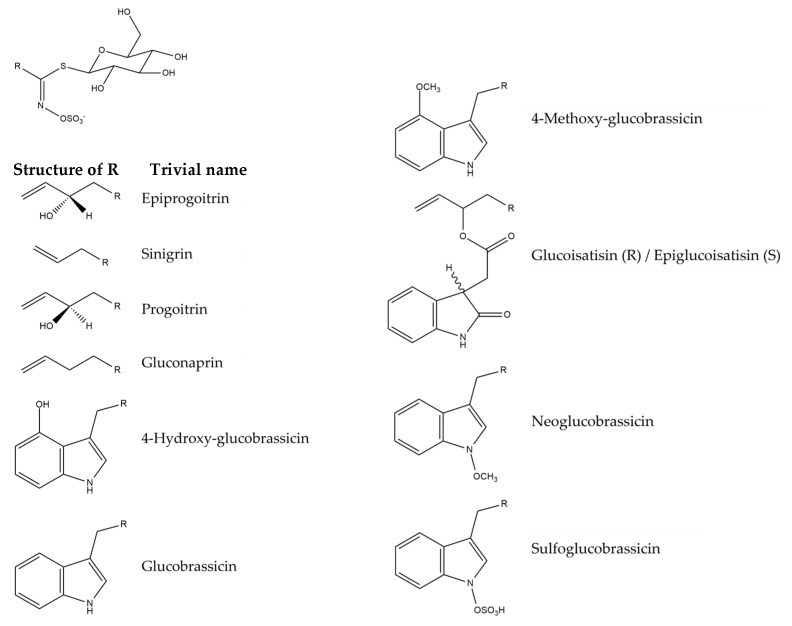
Structures of the glucosinolate compounds in *Isatis tinctoria* seeds [35].

*Isatis tinctoria* contains 10 glucosinolates described in the literature (Figure 7). *Isatis indigotica* contains the same glucosinolates but in different amounts.

When plants are damaged, for example by chewing, they secrete an enzyme called myrosinase, which breaks down glucosinolates into bioactive compounds such as isothiocyanates, thiocyanates, and nitriles. These breakdown products are responsible for the antioxidant, anticarcinogenic, and antimicrobial properties of glucosinolates.

### 7.1. Functions and Properties of Glucosinolates

Glucosinolates play a role in plant defense mechanisms against herbivores, insects, and pathogens, particularly when these metabolites are hydrolyzed by myrosinase [84,85]. The health benefits of consuming plants containing glucosinolates from Brassicaceae are frequently emphasized [86,87,88]. These benefits are related to their antibacterial, antifungal, anticancer, antioxidant, and anti-inflammatory activities [86,87,88,89]. In addition, glucosinolate catabolites, such as isothiocyanates and nitriles, have therapeutic effects in carcinogenesis [89,90]. For instance, by providing chemoprotective effects against carcinogenesis in rat tissues, isothiocyanates offer potential therapeutic benefits for humans [89]. Furthermore, allyl isothiocyanates derived from the biocatalyzed hydrolysis of glucosinolates exhibit antimicrobial activities against specific bacteria [87,91]. From a cosmetic point of view, the antioxidant and anti-inflammatory properties of glucosinolates and their derivatives could help to improve tissue damage induced by oxidative stress and attenuate DNA damage caused by UVB rays, thereby limiting skin aging. Table 11 summarizes the pharmaceutical properties of glucosinolates, but no cosmetic properties are mentioned due to a lack of information.

### 7.2. Sample Preparation, Extraction and Analyses of Glucosinolates

Few methods for extracting and analyzing glucosinolates are described in studies providing the glucosinolate content in woad seeds. These methods and, to a lesser extent, complementary ones will therefore be considered.

Mohn and Hamburger [35] and Angelini et al. [32], who studied the glucosinolate content in seeds of *Isatis tinctoria* and *Isatis indigotica*, respectively, did not provide any information regarding seed preparation and storage conditions. However, it has been noticed that freeze-drying of the raw material is recommended for long-term storage to maintain the recovery yield of glucosinolates [101].

Although several techniques for extraction glucosinolates are described in the literature, only the Soxhlet [32] and ASE [35] methods were chosen by the authors, who provided the glucosinolate contents mentioned in this study.

As mentioned above, Soxhlet extraction is a conventional method that recycles extractive solvent, but it is also time-consuming (2 h–8 h). This method therefore requires high to moderate energy consumption due to the steady and prolonged, but not excessive, heating of the solvent [102].

The unconventional ASE method is advantageous in terms of time, solvent consumption, energetic efficiency, and selectivity of glucosinolate extraction. However, this innovative process is quite expensive and/or not currently suitable for large-scale industrialization [101]. The ASE method uses high temperatures (35 to 200 °C) and high pressures to accelerate the extraction process from woad seeds and improve recovery yields [103]. This results in relatively high energy consumption due to the need to maintain heat and pressure. It is not advisable to extract glucosinolates above 50 °C, as they are thermosensitive and could therefore be degraded, as could their biological efficiency (notably their antioxidant and antimicrobial properties) [102]. ASE does not offer any significant advantage in terms of recovery yield compared to conventional maceration extraction and is not suitable for industrial-scale production. Glucosinolates recovered by ASE have lower biological activities compared to those obtained by other types of extraction, particularly with regard to their inhibition of bacterial growth [101].

It should be noted that many other extraction techniques could have been described, such as reflux set-up [104,105], UAE [101,106], or Supercritical Fluid Extraction (SFE) [107], in order to observe differences in efficiency. Also, glucosinolates must be efficiently separated from other water-soluble compounds in the sample, such as proteins or phenolic compounds.

On the other hand, in terms of energy costs, a ranking of all these extraction methods in ascending order can be suggested: (1) Soxhlet; (2) reflux set-up; (3) UAE; (4) ASE; (5) SFE. Thermal and pressure-based methods, such as ASE and SFE, have significantly higher energy costs than room-temperature methods such as maceration or those using ultrasound activation. Indeed, the choice of an extraction method depends on specific requirements in terms of extraction efficiency, the nature of the compounds to be extracted, and energy and economic costs.

The isolation of glucosinolates from aqueous plant extracts is a challenging process due to their hydrophilic nature, which Mohn and Hamburger [35] and Angelini et al. [32] did not put forward [101]. To overcome this problem, the remedies for purifying glucosinolates include ion-exchange resin-based methods and chromatographic techniques. The approach highlighted by Thies [108] and Wang et al. [106] emphasized ion-exchange resin-based methods, which aim to trap glucosinolates to purify them, thanks to their ionized nature. Ion-exchange resin-based methods, like batch adsorption, are simpler, faster, and more suited to industrial scales, but they do not easily allow the separation of the various glucosinolates in the extract.

On the other hand, chromatographic separation methods aim to isolate the integrality of glucosinolates from plant materials [101]. Preparative High-Performance Liquid Chromatography (prep-HPLC) and High-Speed Counter-Current Chromatography (HSCCC) can be used to isolate pure glucosinolates from Brassicaceae [109,110]. Chromatographic techniques are effective for isolating individual glucosinolates of high purity, but they require precise and expensive equipment, and also consume large amounts of solvents, energy, and time.

The choice of isolation method depends on the intended downstream applications for the isolated glucosinolates [101]. For instance, if the goal is to study the properties of an individual glucosinolate, a chromatographic technique may be preferred. However, if the aim is to obtain a large quantity of glucosinolates for general use, an ion-exchange resin-based method may be more practical. In summary, the isolation of glucosinolates requires a thorough study of their properties and the specific needs of the application. Technological and methodological advances continue to improve the efficiency and selectivity of glucosinolates isolation, making it a rapidly evolving field of research and development.

After being extracted from plant tissues and partially purified, glucosinolates can be identified and quantified, in particular by High-Performance Liquid Chromatography (HPLC). The authors, who quantified glucosinolates in the seeds of *Isatis tinctoria* and *Isatis indigotica*, used HPLC coupled with a Diode Array Detector (HPLC-DAD) [32] and HPLC-MS [35] techniques.

Many other analytical techniques could have been described for qualitative analysis, such as X-ray analysis [111,112], Fourier-transform infrared spectroscopy [113,114], Near-InfraRed Spectroscopy (NIRS), Nuclear Magnetic Resonance spectrometry (NMR) [115,116], ion-exchanger cartridge (desulfation process) [117,118,119], TLC, reversed-phase Ion-Pair Chromatography (IPC) for apolar glucosinolates, and normal-phase HydrophILic Interaction Chromatography (HILIC) for polar glucosinolates. To conclude, the efficiency of the entire extraction process depends on the accurate identification of glucosinolates. To meet the required criteria, a comprehensive characterization must include at least ^1^H NMR, mass spectrometry (MS), and infrared (IR) analyses. Further spectroscopic proofs, such as ^13^C NMR, MS-MS, and elemental analysis, clearly provide crucial structural information on the glucosinolate of interest.

Other methods less described in the literature can also be used to quantify glucosinolates and their breakdown products, as summarized by Śmiechowska et al. [120]. Indeed, some of these methods are based on measuring the glucose or sulfates released following the treatment of glucosinolates by myrosinase. Other methods, such as the glucose test, gravimetric methods, titrimetric methods, Enzyme-Linked ImmunoSorbent Assay (ELISA), GC (after derivatization), Supercritical Fluid Chromatography (SFC) (breakdown products), or Capillary Electrophoresis (CE) (both glucosinolate and their breakdown products) can also be used to quantify these molecules [120]. Furthermore, the amount of glucosinolates can be calculated by various HPLC dosages methods, such as (1) HPLC-UV methods for total glucosinolates [102,121] (at 229 nm and 420 nm) and desulfated glucosinolates [122,123,124] (at 230 and 229 nm); (2) LC-MS/MS methods for desulfated glucosinolates [125,126] and glucosinolates [127]; (3) HPLC-UV regression model for total glucosinolates (at 425 nm) [128].

Finally, HPLC-UV and LC-MS/MS methods are the most commonly used, despite the high costs associated with equipment acquisition and maintenance. Moreover, pure glucosinolate standards are required for accurate LC quantification, but these are not always readily available. Thus, these methods allow for accurate analysis but are not always the most practical ones. In addition, some of the methods mentioned above involve desulfation, which is controversial and requires time-consuming steps. Furthermore, spectrophotometric dosages, and especially the predictive formulas associated with them, offer both simplicity and cost savings.

### 7.3. Glucosinolate Composition

Glucosinolates are sulfur-containing glucose derivatives found in many plant families, especially in the Brassicaceae family, which includes *Isatis tinctoria* and *Isatis indigotica*. Table 12 classifies glucosinolate compounds according to their quantity and collection site. 

The main glucosinolates in *Isatis tinctoria* seeds are aliphatic ones, and especially epiprogoitrin [32,35]. *Isatis indigotica* also has more aliphatic glucosinolates than indolic ones. Its main compound is either progoitrin or epiprogoitrin, depending on the collection site. On the other hand, *Isatis tinctoria* contains more glucosinolates overall than *Isatis indigotica*. 

## 8. Antioxidant Potential

The antioxidant potential of *Isatis tinctoria* has been studied with regards to its oilseed cakes. The raw materials underwent controlled loss-on-drying of less than 10% and were then ground. The hexane extracts were obtained directly from the raw material and all other extracts were defatted with hexane before polar extractions.

Peschel et al. [129] conducted several antioxidant tests on numerous oilseed cakes and plant antioxidants. These samples were compared in Table 13 to a synthetic antioxidant standard (butylated hydroxytoluene or BHT) and three commercial samples of well-established plant antioxidants (grape seed extract, rosemary superfluid extract and green tea extract).

The different assays were undertaken as follows. The Total Phenolic Content (TPC) was studied with the Folin–Ciocalteu reagent and gallic acid as the standard phenolic molecule. The equivalent in µg gallic acid (GAE) of a sample is determined by spectrophotometry (no wavelength described). The free radical scavenging activity (DPPH assay) uses the 1,1-diphenyl-2-picryl-hydrazil (DPPH) reagent and a spectrophotometric determination at 517 nm. The activity is calculated as a percentage of inhibition compared to the blank control. The superoxide anion scavenging activity (NTZ assay) is determined with the nitroblue tetrazolium (NTZ) reagent. The percentage of inhibition is also calculated spectrophotometrically at 560 nm. The Rancimat assay evaluates the inhibition of the oxidation rate of stressed linoleic acid by colorimetric detection, after the oxidation and heating of the samples.

For *Isatis tinctoria*, the most effective extraction in terms of TPC was achieved with ethanol 75%, with 106.10 mg/GAE g.

The radical scavenging activity was investigated with the DPPH assay for free radical scavenging activity and the NTZ assay for superoxide anion scavenging activity. The decolorizing process during the DPPH assay was monitored, and woad extracts with propylene glycol appear to slightly reduce free radicals at a concentration of 10 µg/mL. According to the NTZ assay results, the activities of the polar woad extracts are significantly higher than the commercial rosemary extract (4.30%).

The Rancimat^®^ assay is an accelerated aging test that highlights the oxidation stability of fats and oils. The focus was therefore on the inhibition of linolenic acid oxidation, revealing an inhibition of 30.07%. Also, this assay indicates that the woad extract with propylene glycol has better antioxidant activity than the rosemary standard.

Finally, woad seed extracts, and particularly the propylene glycol extracts, demonstrate interesting antioxidant activity. The toxicity of these extracts could be tested for high-value-added applications, such as in the cosmetics or pharmaceutical industries.

## 9. Conclusions

The review presented *Isatis tinctoria*, especially its fruits and seeds, from a phenological and botanical perspective. This biennial species produces fruits composed of three parts: the pod, the seed, and the pericarp. The present study mainly focused on seeds and only briefly examined these three fruit sections due to a lack of information. A complementary study based on the different parts of the fruit could be interesting to better understand the distribution of molecules.

Since *Isatis tinctoria* species and *Isatis indigotica* species had long been described as the same, comparing their phytochemical composition was relevant. Although the two species were ultimately dissociated, they exhibit obvious phytochemical similarities. Indeed, most of the fatty acids, amino acids, and glucosinolates described in *Isatis tinctoria* have also been described in *Isatis indigotica*. However, *Isatis indigotica* contains additional molecules described in these first two molecular families. Further study of the fruits and seeds of woad could determine whether these additional molecules are also present in *Isatis tinctoria* seeds, or whether they are specific to *Isatis indigotica* seeds. For the time being, these few molecules remain only hypothetical in terms of the composition of woad seeds. On the other hand, four molecular families are described in woad seeds: fatty acids, amino acids, phytosterols and glucosinolates. Also, as some phenolic compounds have been described in *Isatis indigotica* seeds and most plants contain these metabolites, the authors suggest that further studies on woad seeds could focus on this molecular family among others that have not yet been documented. This line of research could be an opportunity to broaden knowledge about this little-described fruit plant.

In addition, numerous cosmetic properties and, to a lesser extent, pharmaceutical and food properties have been summarized. Once their non-toxicity has been verified, woad seeds could be used in these fields of application. However, as the hulling of woad fruit is a very meticulous and time-consuming process, its use would be better suited to high value-added applications.

Furthermore, extraction and analysis methods have been studied for each molecular family. Although these processes are readily available for the study of these compounds, the authors wanted to focus on the energy consumption and efficiency of these techniques, in order to better align with green chemistry.

In addition, the compound content of woad seeds was examined by cross-referencing available sources. This study highlighted the main compounds of *Isatis tinctoria* and compared their concentrations with those of another *Isatis* species. Thanks to this comparison, it is possible to focus on one species or another, depending on the compound or family of compounds targeted.

Finally, the antioxidant activity of woad oilseed cakes has been demonstrated. Once the toxicity issues have been resolved, these antioxidant activities could potentially be exploited in the cosmetics and pharmaceutical industries. Also, a complementary study on the antioxidant activity of whole fruit, seed, oil and pericarp could be relevant. This supplemental study could also link antioxidant activity to single molecules or to a symbiotic group of molecules.

## Figures and Tables

**Table 1:** International names of *Isatis tinctoria* L.

Languages	Vernacular Names	References
None	*Isatis*	[23]
French	Waide Vouède Herbe du Lauraguais Indigo français Indigo des teinturiers	[23]
	Pastel des teinturiers Guède (guedde or guesde) Herbe de Saint-Philippe	[23,24]
	Pastel	[25]
English	Dyer’s woad	[23]
	Woad	[23,24]
German	Waid	[23]
	Färberwaid	[23,25]
Dutch	Wede	[23]
Flemish	Weede	[23]
Italian	Glasto commune (*Glastum* in latin) Guado	[23,25]
Spanish	Guasto Glasto Hierba pastel	[23]
Russian	Ijenack	[23]
Polish	Nilo	[23]
Chinese	Ban Lan Gen (roots) * Da Qing Ye (leaves) * Qing Dai (blue powder)	[23]

* Refer predominantly to *Isatis indigotica*.

**Table 2:** Phenology and germination of *Isatis tinctoria*.

Characteristics	Comments	References
Plant life expectancy	Biennial or short-lived perennial species. (Figure 2)	[3,10,22,29]
First year of growing	Plant becomes a rosette.	[12]
Second year of growing	Stalk grows with flowers. Plant produces seeds before dying.	[12,19]
Dormancy periods	In summer under dry conditions and in winter under cold ones.	[20]
Dormancy duration	Indehiscent seeds. Seeds can survive eight years or longer. Germination rate decreases every year.	[1,19]
Dormancy inhibition	Water-soluble germination inhibitor in silicles removable by leaching. No long dormancy for seeds in growing lands.	[1,12]
Seed germination period	After a period of dormancy, the seeds germinate in the spring or fall.	[12,19]
Influence of sowing period	Stable seed production periods whatever the sowing period (spring or summer).	[4]

**Table 3:** Fatty acid, amino acid, phytosterol and glucosinolate composition of *Isatis tinctoria* and *Isatis indigotica* seeds.

Chemical Classes	*Isatis tinctoria*		*Isatis indigotica*	
	Compounds	References	Compounds	References
Fatty acids	Palmitic acid C16:0 Stearic acid C18:0 Arachidic acid C20:0 Behenic acid C22:0 Palmitoleic acid C16:1∆^9^ Oleic acid C18:1∆^9^ Gondoic acid C20:1∆^11^ /Gadoleic acid C20:1∆^9^ Erucic acid C22:1∆^13^ Linoleic acid C18:2∆^9,12^ Linolenic acid C18:3∆^9,12,15^	[9,29,30]	Palmitic acid C16:0 Stearic acid C18:0 Arachidic acid C20:0 Behenic acid C22:0 Lignoceric acid C24:0 Palmitoleic acid C16:1∆^9^ Oleic acid C18:1∆^9^ Elaidic acid C18:1∆^9^ Vaccenic acid C18:1∆^11^ Linoleic acid C18:2∆^9,12^ Linolenic acid C18:3∆^9,12,15^ Gadoleic acid C20:1∆^9^ Erucic acid C22:1∆^13^ Nervonic acid C24:1∆^15^ 11,14 Eicosadienoic C20:2∆^11,14^ Arachidonic acid C20:4∆^5,8,11,14^	[31,32]
Amino acid	Hydroxyproline Alanine Arginine Aspartic acid Cystine Glutamic acid Glycine Histidine Isoleucine Leucine Lysine Methionine Phenylalanine Proline Serine Threonine Tyrosine Valine	[33]	Alanine Arginine Asparagine Citrulline Cysteine Glutamic acid Glutamine Glycine Histidine Isoleucine Leucine Lysine Methionine Ornithine Phenylalanine Proline Serine Threonine Tryptophan Tyrosine Valine	[34]
Phytosterols	β-Sitosterol Campesterol Δ5-Avenasterol Stigmasterol Brassicasterol Δ7-Avenasterol Δ7-Stigmastenol	[9]	No phytosterols described	/
Glucosinolates	Epiprogoitrin Progoitrin Epiglucoisatisin Glucoisatisin Gluconapin Glucobrassicin 4-Hydroxy-glucobrassicin Neoglucobrassicin	[35]	Epiprogoitrin Progoitrin Epiglucoisatisin Glucoisatisin Gluconapin Glucobrassicin 4-Hydroxy-glucobrassicin Neoglucobrassicin	[32]
Flavonoids	No phenolic compounds described	/	Taxifolin Cyanidin 3-glucoside Kaempferide Quercetin Astragalin Isovitexin 2″-O-beta-D-glucoside 1-O-Galloyl-beta-D-glucose (-)-Epigallocatechin Quercetin 3-beta-D-sophoroside Luteolin 7-glucoside Isoquercitrin Isorhamnetin Luteolin Naringenin Epicatechin Catechin Glycitein Procyanidin B2 Fisetin Eriodictyol	[34]

The nomenclature of fatty acids *Cx:y* Δ^z^ indicates the number of carbons (*Cx*), the number of unsaturation (*y*), the position of the first unsaturation starting from the acid group (*z*).

**Table 4:** Industrial properties and applications of fatty acids contained in *Isatis tinctoria* seeds.

Chemical Classes	Compounds	Applications	Properties	References
Fatty acids —SFA	**Palmitic acid C16:0**	Cosmetic uses	Skin emollient; surfactant—emulsifying agent	[41]
		Cosmetic uses: leave-on products (eye makeup preparations, lipsticks, fragrance products); skin care products; rinse-off products (skin cleansing, shaving cream)	Fragrance agent; opacifying agent; surfactant—cleansing agent; surfactant—emulsifying agent	[42]
	**Stearic acid C18:0**	Cosmetic uses	Cleansing agent; fragrance agent; emulsion stabilizer; surfactant—emulsifying agent; surfactant—cleansing agent	[41]
		Cosmetic uses: leave-on products (eye makeup preparations, eyebrow pencil, eyeliners); skin care products; rinse-off products (bath soaps, detergents, shaving cream); cosmetic sprays (face, neck)	Fragrance agent; surfactant—cleansing agent; surfactant—emulsifying agent	[42]
		Pharmaceutical uses: suppositories; coating enteric pills; ointments; coating bitter remedies; stearin soap (for opodeldoch)	/	
	**Arachidic acid C20:0**	Cosmetic uses	Cleansing agent; opacifying agent; surfactant—emulsifying agent; surfactant—cleansing agent	[41]
		Cosmetic uses	Opacifying agent; surfactant—cleansing agent	[42]
	**Behenic acid C22:0**	Cosmetic uses	Cleansing agent; opacifying agent; surfactant—emulsifying agent; surfactant—cleansing agent	[41]
		Cosmetic uses: lipstick, eyebrow pencil	Opacifying agent; surfactant—cleansing agent	[42]
Fatty acids —MUFA	**Palmitoleic acid C16:1 ∆^9^**	Cosmetic uses	Not referenced	[41,42]
	**Oleic acid C18:1 ∆^9^**	Cosmetic uses	Skin emollient; surfactant—emulsifying agent	[41]
		Cosmetic uses: spray deodorants	Fragrance agent; surfactant—cleansing agent	[42]
		Pharmaceutical uses: emulsifying and solubilizing agent (in pharmaceutical acids); diagnostic aid (for pancreatic function); ointments	/	
		Pharmaceutical uses	Cholesterol stabilizer	[43,44]
	**Gadoleic acid C20:1∆^9^**	Cosmetic uses	Not referenced	[41,42]
	**Gondoic acid C20:1∆^11^**	Cosmetic uses	Hair conditioner	[41]
	**Erucic acid C22:1 ∆^13^**	Cosmetic uses	Skin care agent	[41]
		Cosmetic uses	Skin-conditioning agent—miscellaneous	[42]
Fatty acids —PUFA	**Linoleic acid C18:2 ∆^9,12^**	Cosmetic uses	Antistatic agent; skin care agent; cleansing agent; hair conditioning agent; skin emollient; surfactant—cleansing agent	[41]
		Cosmetic uses: leave-on skin care products; rinse-off skin cleansing products; skin care products (face, neck, body, hand)	Fragrance agent; hair conditioning agent; skin conditioning agent—miscellaneous; surfactant—cleansing agent	[42]
		Pharmaceutical uses: vitamins	/	
		Pharmaceutical uses	Cholesterol stabilizer	[43,45,46]
	**Linolenic acid C18:3 ∆^9,12,15^**	Cosmetic uses	Antistatic agent; skin care agent; cleansing agent; fragrance agent; hair conditioning agent; skin emollient; surfactant—cleansing agent	[42]
		Cosmetic uses	Fragrance agent; hair conditioning agent; skin conditioning agent—miscellaneous; surfactant—cleansing agent	
		Food uses: dietary supplement/nutrient	/	
		Pharmaceutical uses	Cholesterol stabilizer	[43,47,48]

Note 1. European Food Safety Authority (EFSA) also provided more general content on MUFAs and PUFAs and their contribution to the maintenance of normal blood cholesterol levels [48,49].

**Table 5:** Fatty acid composition of *Isatis tinctoria* and *Isatis indigotica* seeds at harvest.

Chemical Classes	Compounds	*Isatis tinctoria*			*Isatis indigotica*		
		Quantities	Sites of Collection	References	Quantities	Sites of Collection	References
Fatty acids —SFA	**Palmitic acid** **C16:0**	5.07 11.18 6	France ^1^ Turkey ^2^ USA ^3^	[9] [29] [51]	4.59 ^a^/4.91 ^b^ 4.4 ^c^/5.3 ^d^/4.5 ^e^/5.1 ^f^	China ^4^ Italy ^5^	[31] [32]
	**Stearic acid** **C18:0**	2.35 2.17 2	France ^1^ Turkey ^2^ USA ^3^	[9] [29] [51]	2.1 ^c^/1.5 ^d^/1.4 ^e^/1.7 ^f^	Italy ^5^	[32]
	**Arachidic acid** **C20:0**	1.68 1.22 2	France ^1^ Turkey ^2^ USA ^3^	[9] [29] [51]	1.33 ^a^/1.28 ^b^ 1.5 ^c^/1.1 ^d^/1.0 ^e^/1.3 ^f^	China ^4^ Italy ^5^	[31] [32]
	**Behenic acid** **C22:0**	0.48 0.66 Trace	France ^1^ Turkey ^2^ USA ^3^	[9] [29] [51]	0.7 ^c^/0.5 ^d^/0.4 ^e^/0.7 ^f^	Italy ^5^	[32]
	**Lignoceric acid C24:0**	/	/	/	0.5 ^c^/0.4 ^d^/0.4 ^e^/0.4 ^f^	Italy ^5^	[32]
	***Total SFA***	***9.58*** ***15.23*** ***10***	France ^1^ Turkey ^2^ USA ^3^	[9] [29] [51]	***5.92 ^a^/6.19 ^b^*** ***9.2 ^c^/8.8 ^d^/7.7 ^e^/9.2 ^f^***	China ^4^ Italy ^5^	[31] [32]
Fatty acids —MUFA	**Palmitoleic acid** **C16:1 ∆^9^**	0.180	France ^1^ USA ^3^	[9] [51]	0.22 ^a^/0.27 ^b^	China ^4^	[31]
	**Oleic acid** **C18:1 ∆^9^**	16.51 14.64 16	France ^1^ Turkey ^2^ USA ^3^	[9] [29] [51]	27.6 ^c^/12.9 ^d^/18.4 ^e^/14.0 ^f^	Italy ^5^	[32]
	**Elaidic acid C18:1** **∆^9^**	n.d.	n.d.	n.d.	20.30 ^a^/19.60 ^b^	China ^4^	[31]
	**Vaccenic acid C18:1∆^11^**	n.d.	n.d.	n.d.	2.0 ^c^/2.2 ^d^/1.2 ^e^/1.4 ^f^	Italy ^5^	[32]
	Gondoic acid C20:1∆^11^ * **/** **Gadoleic acid C20:1** ∆^9^ *	10.01 10.40 13	France ^1^ Turkey ^2^ USA ^3^	[9] [29] [51]	11.89 ^a^/11.32 ^b^ 10.9 ^c^/8.2 ^d^/10.5 ^e^/8.7 ^f^	China ^4^ Italy ^5^	[31] [32]
	**Erucic acid** **C22:1 ∆^13^**	20.30 26.48 20	France ^1^ Turkey ^2^ USA ^3^	[9] [29] [51]	25.00 ^a^/23.81 ^b^ 16.6 ^c^/18.2 ^d^/18.1 ^e^/21.0 ^f^	China ^4^ Italy ^5^	[31] [32]
	**Nervonic acid C24:1∆^15^**	n.d.	n.d.	n.d.	1.4 ^c^/2.2 ^d^/3.3 ^e^/2.1 ^f^	Italy ^5^	[32]
	***Total MUFA***	***47.01*** ***51.52*** ***49***	France ^1^ Turkey ^2^ USA ^3^	[9] [29] [51]	***57.41 ^a^/55.00 ^b^*** ***58.4 ^c^/43.7 ^d^/51.4 ^e^/47.3 ^f^***	China ^4^ Italy ^5^	[31] [32]
Fatty acids —PUFA	**Linoleic acid** **C18:2 ∆^9,12^**	12.40 2.74 12	France ^1^ Turkey ^2^ USA ^3^	[9] [29] [51]	9.75 ^a^/10.44 ^b^ 10.9 ^c^/15.6 ^d^/11.6 ^e^/11.5 ^f^	China ^4^ Italy ^5^	[31] [32]
	**11,14 Eicosadienoic** **C20:2∆^11,14^**	n.d.	n.d.	n.d.	0.5 ^c^/1.0 ^d^/0.8 ^e^/0.8 ^f^	Italy ^5^	[32]
	**Linolenic acid** **C18:3 ∆^9,12,15^**	31.00 14.05 28	France ^1^ Turkey ^2^ USA ^3^	[9] [29] [51]	26.92 ^a^/28.37 ^b^ 19.8 ^c^/28.6 ^d^/26.8 ^e^/29.2 ^f^	China ^4^ Italy ^5^	[31] [32]
	**Arachidonic acid** **C20:4∆^5,8,11,14^**	n.d.	n.d.	n.d.	0.4 ^c^/0.7 ^d^/0.6 ^e^/0.8 ^f^	Italy ^5^	[32]
	***Total PUFA***	**43.41** **16.79** **40**	France ^1^ Turkey ^2^ USA ^3^	[9] [29] [51]	***36.67 ^a^/38.81 ^b^*** ***31.1 ^c^/44.9 ^d^/39.0 ^e^/41.5 ^f^***	China ^4^ Italy ^5^	[31] [32]
	***Total fatty acids***	***100.00*** 86.84 ** ***99***	France ^1^ Turkey ^2^ USA ^3^	[9] [29] [51]	***100 ^a^/100 ^b^*** 99.6 * ^c^/99.1 * ^d^/ 99.1 * ^e^/99.2 * ^f^	China ^4^ Italy ^5^	[31] [32]
Oil	***Oil content (%)***	***16.09*** ***10.0*** ***13***	France ^1^ Turkey ^2^ USA ^3^	[9] [29] [51]	***37.35 ^c^/37.60 ^d^/*** ***36.40 ^e^/35.85 ^f^***	Italy ^5^	[32]
Protein	***Protein content (%)***	***12***	USA ^3^	[51]	***36.33 ^c^/36.49 ^d^/*** ***36.84 ^e^/36.46 ^f^***	Italy ^5^	[32]

The quantity values of SFAs (saturated fatty acids), MUFAs (monounsaturated fatty acids) and PUFAs (polyunsaturated fatty acids) are expressed in % of the DM. ^1^ CAPA Institute (Coopérative Agricole des Plaines d’Ariège), France. ^2^ Seed Collection unit of Medicinal and Aromatic Plants, Department of field Crops, faculty of Agriculture, Dicle University, Diyarbakir, Turkey. ^3^ Northern Regional Research Laboratory, Peoria, Illinois, USA. ^4^ Shaanxi Geo-authentic Medicinal Plant Co. Ltd., Xi’an, China. ^5^ Four *Isatis indigotica* accessions (II1, II2, II3, II4), originating from China, were studied under field conditions at San Piero a Grado (Tuscany region, Central Italy, 43°40′ N latitude; 10°19′ E longitude) in 2010 growing season. ^a^ Soxhlet extraction. ^b^ Ultrasound-assisted extraction. ^c^ Accession II 1. ^d^ Accession II 2. ^e^ Accession II 3. ^f^ Accession II 4. n.d. = Not detected. * The identification of the C20:1 fatty acid is confusing for *Isatis tinctoria*. Roche et al. [9] identified this molecule as cis-9-Eicosenoic acid, Kizil et al. [29] as 11-Eicosenoic acid and Mikolajczak et al. [51] did not provide an identifying name. Either an identification error has been made in one of these studies, or both molecules are present. However, Angelini et al. [32] and Li et al. [31] both identified gadoleic acid in *Isatis indigotica*. ** In the study, the total fatty acid content is calculated with additional fatty acids not mentioned in the present review. Note 1. The reference [9] uses isolated seeds, the reference [51] uses seed and pericarp together, and the other reference [29] is not clear if the material is seed and pod together. Note 2. Dolya et al. [30] also highlighted fatty acid content, but data are not represented here because no experimental details are provided.

**Table 6:** Physico-chemical characteristics of *Isatis tinctoria* and *Isatis indigotica* oils.

Physico-Chemical Characteristics	*Isatis tinctoria* Oil		*Isatis indigotica* Oil
	Ref. [30] ^1^	Ref. [51] ^2^	Ref. [31] ^3^
Density $\left(d_{4}^{20}\right)$	0.9187	n.d.	n.d
Refractive index $\left(n_{D}^{20}\right)$	1.4760	n.d.	1.4725 ^a^/1.4732 ^b^
Relative viscosity $\left(E_{20}^{0}\right)$	9.85	n.d.	n.d.
Acid index (mg KOH/g)	1.19	n.d.	2.09 ^a^/2.36 ^b^
Saponification index (mg KOH/g)	177.58	n.d.	171.00 ^a^/174.68 ^b^
Ester index (mg KOH/g)	176.39 *	171 *	168.91 ^a^*/172.32 ^b^*
Peroxide index (meq O_2_/kg)	n.d.	n.d.	5.48 ^a^/5.37 ^b^
Iodine index (%)	130.48	136	105.41 ^a^/102.60 ^b^
MW_FA_ (g/mol)	947.836468	984.308772	963.572246
Unsaponifiable (%)	1.86	n.d.	n.d.
Phosphatides (%)	0.53	n.d.	n.d.

^1^ Zaporozhe Medical Institute, Ukraine. ^2^ Northern Regional Research Laboratory, Peoria, Illinois, USA. ^3^ Shaanxi Geo-authentic Medicinal Plant Co. Ltd., Xi’an, China. ^a^ Soxhlet extraction. ^b^ Ultrasound-assisted extraction. n.d. = Not determined. * Data calculated by the authors of the present review. Note 1. Dolya et al. [30] provided data without experimental details. Note 2. Mikolajczak et al. [51] only provided iodine index and MW_FA_ values.

**Table 7:** Industrial properties and applications of the amino acids contained in *Isatis tinctoria* seeds.

Compound	Applications	Properties	References
**Alanine**	Cosmetic uses	Antistatic agent; skin care agent; fragrance agent; hair conditioner	[41]
**Arginine**	Cosmetic uses	Antistatic agent; skin care agent; fragrance agent; hair conditioner	[41]
**Aspartic acid**	Cosmetic uses	Antistatic agent; skin care agent; fragrance agent; hair conditioner	[41]
**Cystine**	Cosmetic uses	Antistatic agent; fragrance agent; hair conditioner; humectant	[41]
**Glutamic acid**	Cosmetic uses	Antistatic agent; hair conditioner,	[41]
**Glycine**	Cosmetic uses	Antistatic agent; skin care agent; buffer agent; hair conditioner	[41]
**Histidine**	Cosmetic uses	Antistatic agent; skin care agent; humectant	[41]
**Hydroxyproline**	Cosmetic uses	Antistatic agent; skin care agent; hair conditioner; surfactant—cleanser	[41]
	Pharmaceutical uses	Pharmaceutical synthesis reagent; potential anti-cancer agent	[63]
**Isoleucine**	Cosmetic uses	Antistatic agent; skin care agent; hair conditioner	[41]
**Leucine**	Cosmetic uses	Antistatic agent; skin care agent; hair conditioner	[41]
**Lysine**	Cosmetic uses	Antistatic agent; skin care agent; hair conditioner	[41]
**Methionine**	Cosmetic uses	Antistatic agent; skin care agent; hair conditioner	[41]
**Phenylalanine**	Cosmetic uses	Skin care agent; fragrance agent; hair conditioner	[41]
**Proline**	Cosmetic uses	Skin care agent; hair conditioner	[41]
**Serine**	Cosmetic uses	Antistatic agent; skin care agent; fragrance agent; hair conditioner	[41]
**Threonine**	Cosmetic uses	Antistatic agent; curl or stretch agent; hair conditioner	[41]
**Tyrosine**	Cosmetic uses	Antistatic agent; skin care agent; fragrance agent; hair conditioner	[41]
**Valine**	Cosmetic uses	Antistatic agent; skin care agent; fragrance agent; hair conditioner	[41]

**Table 8:** Amino acid composition of *Isatis tinctoria* and *Isatis indigotica* seeds at harvest.

Chemical Classes	Comments/Compounds	Quantities	
		*Isatis tinctoria* [33] ^1^	*Isatis indigotica* [34] ^2^
**Oil** ^a^	^b^	12.6% ^c^	n.d.
**Proteins**	In whole seed ^a,b^	12.5% ^c^	n.d.
	In extracted meal ^a,b^	14.3% ^c^	n.d.
**Amino acids**	Nitrogen distribution as % of total nitrogen	69.8% ^c^	n.d.
	Alanine Arginine Aspartic acid Cystine Glutamic acid Glycine Histidine Hydroxyproline Isoleucine Leucine Lysine Methionine Phenylalanine Proline Serine Threonine Tyrosine Valine	232 377 421 148 872 315 139 148 211 370 327 102 233 346 225 212 137 283	n.d. p. ^d^/n.p. ^e^ p. ^d^/n.p. ^e^ n.p. ^d^/p. ^e^ p. ^d^/n.p. ^e^ n.d. n.p. ^d^/p. ^e^ n.d. p. ^d^/n.p. ^e^ p. ^d^/n.p. ^e^ n.p. ^d^/n. ^e^ p. ^d^/n.p. ^e^ n.p. ^d^/p. ^e^ p. ^d^/n.p. ^e^ p. ^d^/n.p. ^e^ p. ^d^/n.p. ^e^ p. ^d^/n.p. ^e^ n.d.

The values relating to amino acids quantities are expressed in milligrams of amino acid per gram of nitrogen for reference [33]. For reference [34], no quantitative values were described, except for the presence rate of the amino acids following fruit maturation. ^1^ Collected by Crops Research Division, Agricultural Research Service, U.S. Department of Agriculture, Beltsville, Md, USA. ^2^ Greenhouse at Huaihua University (27330 3900N, 109590 0800E), Hunan Province, China. ^a^ On dry basis. ^b^ After acid hydrolysis. ^c^ %N × 6.25. ^d^ Beginning of maturation (30 days after flowering). ^e^ End of maturation (51 days after flowering). n.d. = not described; n.p. = not present; p. = present; n. = neutral status.

**Table 9:** Industrial properties and applications of phytosterols contained in *Isatis tinctoria* seeds.

Compounds	Applications	Properties	References
**β-Sitosterol**	Cosmetic uses	Skin care agent; fragrance agent; light stabilizing agent; emulsion stabilizer	[41]
	Cosmetic and pharmaceutical uses	Antioxidant; antimicrobial; angiogenic; immunomodulatory; antidiabetic; anti-inflammatory; anti-cancer; antinociceptive	[78]
	Cosmetic uses	Fragrance agent; skin-conditioning agent—miscellaneous	[79]
	Food uses: food products (heart health benefits)	Coronary heart disease risk reducer	
**Campesterol**	No cosmetic uses referenced	No cosmetic properties referenced	[41]
	Pharmaceutical uses	Alleviator of arthritis symptoms ^1^	[80]
	Cosmetic uses	Skin-conditioning agent—emollient (in mixture of phytosterols obtained from rapeseed)	[79]
	Food uses: food products (heart health benefits)	Coronary heart disease risk reducer	
**Δ^5^-Avenasterol**	No cosmetic uses referenced	No cosmetic properties referenced	[41,79]
	Cosmetic uses	Antioxidant	[68]
	Cosmetic and pharmaceutical uses	Cholesterol-lowering effector; cardiovascular diseases reducer; anti-inflammatory	[73]
**Stigmasterol**	No cosmetic uses referenced	No cosmetic properties referenced	[41]
	Cosmetic and pharmaceutical uses	Anti-inflammatory; antidiabetic; antioxidant; cholesterol-lowering effector; anti-tumoral	[81]
	Cosmetic uses	Skin-conditioning agent—emollient (in mixture of phytosterols obtained from soybean)	[79]
	Food uses: food products (heart health benefits)	Coronary heart disease risk reducer	
**Brassicasterol**	No cosmetic uses referenced	No cosmetic properties referenced	[41]
	Pharmaceutical uses	Nutritional and biological values; anti-infector ^2^; cardiovascular diseases reducer	[82]
	Cosmetic uses	Skin-conditioning agent—emollient (in mixture of phytosterols obtained from rapeseed)	[79]

^1^ The molecule seems to be effective in the expression of pro-inflammatory modulation of cytokines, and anti-inflammatory cytokines, hence posing therapeutic potential in Rheumatoid Arthritis management. ^2^ Acts against herpes simplex virus type 1 (HSV-1) and Mycobacterium tuberculosis. No applications or properties have been identified for Δ^7^-Avenasterol and Δ^7^-Stigmastenol trace molecules referenced by Roche et al. [9].

**Table 10:** Phytosterol composition of *Isatis tinctoria* seeds at harvest, collected by the CAPA Institute (Coopérative Agricole des Plaines d’Ariège), France (data from [9]).

Compounds	Quantities
**β-Sitosterol**	68.25 ± 0.12
**Campesterol**	20.32 ± 0.07
**Δ5-Avenasterol**	14.82 ± 0.12
**Stigmasterol**	3.41 ± 0.02
**Brassicasterol**	3.37 ± 0.04
***Total phytosterols (desmethysterols)***	***114.11 ± 0.32***

The values of phytosterols are given in mg/100 g of dry material. The total amount of phytosterols represents only desmethylsterols. Δ^7^-avenasterol and Δ^7^-stigmastenol referenced by Roche et al. [9] were found in trace amounts. No phytosterols are described in the literature for *Isatis indigotica*.

**Table 11:** Industrial properties and applications of glucosinolates contained in *Isatis tinctoria* seeds.

Chemical Classes	Compounds	Applications	Properties	References
	**Epiprogoitrin**	No cosmetic uses referenced	No cosmetic properties referenced	[41]
**Aliphatic glucosinolates**		Pharmaceutical uses: antiviral drug resources (virus strain A/California/7/2009 (H1N1)).	Anti-viral efficacy (*in vitro* and *in ovo*)	[92]
		Pharmaceutical uses: nematode control (*Meloidogyne hapla*)	Inhibition of infectious juveniles	[93]
	**Progoitrin**	No cosmetic uses referenced	No cosmetic properties referenced	[41]
		Pharmaceutical uses: antiviral drug resources (virus strain A/California/7/2009 (H1N1).	Anti-viral efficacy (*in vitro* and *in ovo*)	[92]
	**Gluconapin**	No cosmetic uses referenced	No cosmetic properties referenced	[41]
	**Epiglucoisatisin**	No cosmetic uses referenced	No cosmetic properties referenced	[41]
**Indolic glucosinolates**		Pharmaceutical uses	Potential agent in the treatment of urease- and protease-associated complications	[94]
	**Glucoisatisin**	No cosmetic uses referenced	No cosmetic properties referenced	[41]
	**Glucobrassicin**	No cosmetic uses referenced	No cosmetic properties referenced	[41]
		Pharmaceutical uses	Anti-cancer, antioxidant, antibacterial and anti-inflammatory	[95]
		Pharmaceutical uses: anti-tumoral drug	Tumor inhibition agent	[96,97,98]
		Pharmaceutical/food uses: enrichment of pizza (cauliflower byproduct flours for fortification level)	Chemoprotective agent in pre-clinical models (breakdown products)	[97,99]
		Pharmaceutical/food uses: lowering of blood LDL/VLDL ^1^ cholesterol levels (through the consumption of glucobrassicin-rich vegetables)	Cholesterol-lowering effector	[100]
	**4-Hydroxy-** **glucobrassicin**	No cosmetic uses referenced	No cosmetic properties referenced	[41]
	**Neoglucobrassicin**	No cosmetic uses referenced	No cosmetic properties referenced	[41]

^1^ Low-density lipoprotein (LDL)/very low-density lipoprotein (VLDL).

**Table 12:** Glucosinolates composition of *Isatis tinctoria* and *Isatis indigotica* seeds at harvest.

Chemical Classes	Compounds	Quantities		
		*Isatis tinctoria* [35] ^1^	*Isatis indigotica* [35] ^1^	*Isatis indigotica* [32] ^2^
Aliphatic glucosinolates	**Epiprogoitrin**	130	50	115.42 ^a^/112.90 ^b^/18.56 ^c^/68.77 ^d^
	**Progoitrin**	90	140	28.64 ^a^/27.65 ^b^/64.20 ^c^/34.70 ^d^
	**Gluconapin**	55	10	3.16 ^a^/0.79 ^b^/32.89 ^c^/31.03 ^d^
	***Total aliphatic glucosinolates***	***275***	***200***	***147.22 ^a^/141.34 ^b^/115.65 ^c^/134.5 ^d^***
Indolic glucosinolates	**Epiglucoisatisin** **Glucoisatisin**	90 both isomers together	120 both isomers together	0.95 ^a^/0.65 ^b^/0.53 ^c^/0.44 ^d^
	**Glucobrassicin**	45	n.d.	0.10 ^a^/0.07 ^b^/4.37 ^c^/0.70 ^d^
	**4-Hydroxy-glucobrassicin**	15	10	3.44 ^a^/2.30 ^b^/2.57 ^c^/2.36 ^d^
	**Neoglucobrassicin**	10	n.d.	0.26 ^a^/0.21 ^b^/0.12 ^c^/0.14 ^d^
	***Total indolic glucosinolates***	***160***	***130***	***4.75 ^a^/3.23 ^b^/7.59 ^c^/3.64 ^d^***
	***Total glucosinolates***	***435***	***330***	***151.96 ^a^/144.54 ^b^/123.24 ^c^/138.13 ^d^***

The values of glucosinolates are given in estimated mg/100 g DM for reference [35] and in µmol/g FW for reference [32]. ^1^ Collected by The Agricultural Research Station of Thuringia (TLL), Dornburg, Germany. ^2^ Four *Isatis indigotica* accessions (II1, II2, II3, II4), originating from China, were studied under field conditions at San Piero a Grado (Tuscany region, Central Italy, 43°40′ N latitude; 10°19′ E longitude) in 2010 growing season. ^a^ Accession II 1. ^b^ Accession II 2. ^c^ Accession II 3. ^d^ Accession II 4. n.d. = not detected.

**Table 13:** Yield and activity of *Isatis tinctoria* oilseed cakes in comparison to BHT, grape seed extract, rosemary SF and green tea extract (data from [129]).

		EM (%)	TPC (mg/GAE g)	DPPH (%)	NTZ (%)	Rancimat (%)
*Isatis* *tinctoria* extracts	**Hexane**	9.63	10.91 ± 3.00	2.51 ± 0.19	2.50 ± 0.76	−2.72 ± 2.48 ^1^
	**Water (60 °C)**	32.94	nd	nd	nd	nd
	**Propylene glycol**	10.59	74.61 ± 3.03	7.14 ± 0.75	15.08 ± 2.30	36.03 ± 3.06 ^2^
	**Ethanol (75%)**	13.72	106.10 ± 2.10	6.88 ± 1.12	14.53 ± 2.82	30.07 ± 4.34
	**Isopropanol**	1.12	60.63 ± 0.65	6.08 ± 0.37	10.10 ± 0.47	25.93 ± 0.830
Standard references	**BHT**	/	/	10.56 ± 1.32	19.34 ± 4.70	611.98 ± 37.11
	**Grape seed**	/	790.00 ± 53.08	79.14 ± 0.99	47.46 ± 2.31	0.80 ± 1.18
	**Green tea**	/	446.79 ± 27.40	61.79 ± 1.14	38.34 ± 1.97	247.30 ± 12.78
	**Rosemary**	/	142.10 ± 1.98	19.42 ± 0.19	4.30 ± 8.60	32.28 ± 1.75

DPPH assay concentration of 10 µg/mL; NTZ assay concentration of 20 µg/mL, Rancimat^®^ sample concentration of 0.4 mg/g; nd: not determined. ^1^ Pre-solution of the extracts in soybean oil. ^2^ Direct use of propylene glycol extracts in the assay.
